# Safety and feasibility of transcranial direct current stimulation in end-stage renal disease patients undergoing hemodialysis: an exploratory study

**DOI:** 10.1590/2175-8239-JBN-2024-0010en

**Published:** 2024-07-19

**Authors:** Rodrigo Pegado, Monaliza Melo, Tayanne Oehmen, Gianna Mastroianni Kirsztajn, Edson Silva-Filho, Artur Quintiliano

**Affiliations:** 1 Universidade Federal do Rio Grande do Norte, Programa de Pós-Graduação em Ciências da Reabilitação, Natal, RN, Brazil. Universidade Federal do Rio Grande do Norte Programa de Pós-Graduação em Ciências da Reabilitação Natal RN Brazil; 2 Universidade Federal do Rio Grande do Norte, Programa de Pós-Graduação em Ciências da Saúde, Natal, RN, Brazil. Universidade Federal do Rio Grande do Norte Programa de Pós-Graduação em Ciências da Saúde Natal RN Brazil; 3 Universidade Federal do Rio Grande do Norte, Natal, RN, Brazil. Universidade Federal do Rio Grande do Norte Natal RN Brazil; 4 Universidade Federal de São Paulo, Programa de Pós-Graduação em Nefrologia, São Paulo, SP, Brazil. Universidade Federal de São Paulo Programa de Pós-Graduação em Nefrologia São Paulo SP Brazil; 5 Universidade Federal do Rio Grande do Norte, Departamento de Medicina, Natal, RN, Brazil Universidade Federal do Rio Grande do Norte Departamento de Medicina Natal RN Brazil

**Keywords:** Transcranial Direct Current Stimulation, Renal Dialysis, Arterial Pressure, Heart Rate

## Abstract

**Introduction:**

Patients with end-stage renal disease often face a challenging routine of hemodialysis, dietary restrictions, and multiple medications, which can affect their hemodynamic function. Home-based, safe, and nonpharmacological approaches such as transcranial direct current stimulation (tDCS) should be combined with conventional treatment.

**Objective:**

To assess the safety and feasibility of tDCS on blood pressure and heart rate in patients with end-stage renal disease undergoing hemodialysis.

**Method:**

This is a parallel, randomized, sham-controlled trial. Patients undergoing hemodialysis for more than three months were included. The patients received ten non-consecutive 2mA tDCS sessions on the primary motor cortex . Each session lasted 20 minutes. At baseline and after each of the ten sessions, blood pressure and heart rate of the patients were measured hourly for four hours.

**Results:**

Thirty patients were randomized to the active or sham group. The mean difference between the groups was calculated as the mean value of the sham group minus the mean value of the active group. Despite there were no statistical changes for all outcomes considering all 10 sessions, we found differences between groups for systolic –10.93 (–29.1;7.2), diastolic –3.63 (–12.4; 5.1), and mean blood pressure –6.0 (–16.3; 4.2) and hear rate 2.26 (–2.5; 7.1). No serious adverse events were found. The active group showed higher blood pressure values at all points, while heart rate was lower in the active group.

**Conclusion:**

tDCS is safe and feasible for patients with end-stage renal disease undergoing hemodialysis. Future studies should investigate whether tDCS could potentially induce a hypotensive protective effect during hemodialysis.

## Introduction

End-stage renal disease is a consequence of chronic kidney disease, which is a global problem due to the increasing number of affected individuals and the high cost of treatment^1^. Research estimates that chronic kidney disease affects more than 10% of the world’s population. The incidence of the most severe stages, in which hemodialysis is necessary, is growing annually at a rate of 6–7%^1^. In the severe stage, the patients are exposed to a weekly routine of multiple visits to specialized clinics, medications, and activity limitations. Thus, patients with end-stage renal disease experience chronic pain, significant functional limitations, changes in mental health, and a decrease in overall quality of life^2^.

One of the most common symptoms presented by patients undergoing hemodialysis is hemodynamic oscillations, which are often neglected by the healthcare team^3^. In addition to hemodynamic changes, pain, depression, anxiety, restless leg syndrome, and reduced sleep quality are associated symptoms^4,5^. In this sense, pharmacological and non-pharmacological treatments prevent and reduce physical and behavioral symptoms of patients with end-stage renal disease undergoing hemodialysis^4,5^. However, collateral effects related to medication and dialysis could increase the incidence of comorbidities and death^6,7^.

Recently, Quintiliano et al.^8^ presented a new therapeutic proposal for patients with end-stage renal disease undergoing hemodialysis, aiming to improve pain, mood, and overall physical function. The authors suggested that 10 sessions of 2 mA anodal transcranial direct current stimulation (tDCS) over the primary motor cortex (C3/Fp2 montage) improve pain, depression, anxiety, and quality of life of patients undergoing hemodialysis^8^. tDCS is a noninvasive technique for modulating brain areas related to pain and mood^9,10^. Through a microcurrent flow, a change in the neuronal depolarization capacity and temporary plasticity of neural circuits occurs. Depending on the set-up and intensity, physical and behavioral effects are achieved^10^.

It is important to mention that most studies focused on the hemodynamic response after tDCS in healthy individuals^11,12^. Also, studies did not report the hemodynamic safety of tDCS during clinical procedures, including hemodialysis. Throughout the hemodialysis procedure, hemodynamic parameters can present significant clinical variations and generate adverse effects. Hemodynamic instability during hemodialysis can occur in some patients and is a recognized potential complication of the procedure^13^. Hemodynamic complications are associated with different factors, including changes in fluid and electrolyte balance, alterations in cardiac function, and hypotension^13^.

tDCS emerges as a potential low-cost tool to help patients with end-stage renal disease. It is imperative that tDCS does not interfere negatively with hemodynamic parameters during or after hemodialysis sessions. Besides, tDCS could generate protective effects by preventing hypotension, which is frequently reported by patients during hemodialysis. Therefore, we hypothesize that the application of C3/Fp2 tDCS in end-stage renal disease patients during hemodialysis is safe and feasible, and might prevent hypotensive dysfunctions. Considering these assumptions, the study aims to assess the safety and feasibility of tDCS in patients with end-stage renal disease undergoing hemodialysis and the impact on blood pressure and heart rate.

## Methods

### Study Design

This was a single-center, parallel, randomized, sham-controlled trial, designed in accordance with the Consolidated Standards of Reporting Trials statement^14^, the Declaration of Helsinki, and resolution No. 466/12 of the National Health Council. This study was previously approved by the local Ethics Committee (Faculty of Health Science of Trairí) (number 2715151) and retrospectively registered on the Brazilian Clinical Trials Registry (RBR-46vhrkj). The study was conducted at the Kidney Institute, Natal, Brazil between August 2018 and February 2020. The researchers explained the study’s objective and protocol to all participants who needed to sign the written informed consent to participate.

### Eligibility Criteria

Patients were included in the study according to the following criteria: men or women aged between 18 to 75 years undergoing hemodialysis (four-hour session) for more than three months and presenting chronic musculoskeletal pain, headache, or neuropathic pain (scoring >4 on the visual analog scale for more than three months). Patients were excluded if they had electrical implants in the body, clinical contraindications to receive tDCS, such as having metal embedded in their scalp or brain, a history of epilepsy or convulsion pregnant women, signs of severe disease or indication of hospitalization including previous hemodynamic instability, acute myocardial infarction, infection, stroke, and psychiatric illness.

### Intervention

An experienced and trained nurse applied a total of ten nonconsecutive sessions three times a week (Monday, Wednesday, and Friday or Tuesday, Thursday, and Saturday) to each participant. The patients started the hemodialysis and monitoring in a comfortable chair with back and arm support. At the beginning of hemodialysis, the tDCS was assembled and turned on.

Initially, the electrodes were placed into a 35 cm^2^ sponge hydrated with saline solution (154 mM NaCl, approximately 12 mL per sponge). Then, the anode electrode was positioned and attached by elastic bands over the region of the left primary motor cortex (C3) and the cathode electrode on the right supraorbital region (Fp2), according to the international 10−20 electroencephalography system “M1-SO” assembly. In the active group, the direct current began at an intensity of 2 mA started with a 30-second gradual current ramp-up. After 20 minutes, a 30-second gradual current ramp-down finished the session. The same protocol was used for the Sham group, but the ramp-up and the ramp-down of the current occurred for only 30 seconds^8^. The current was delivered through electrodes by a battery-powered stimulator, and the current was verified with a precision digital multimeter (DT832, WeiHua Electronic Co.,Ltd, China) with a standard error of 1.5% . The appearance of the device was identical in the active and sham settings. For ethical issues, there were no changes in hemodialysis routine (days, time, and place of sessions), medications, laboratory, and image exams.

### Outcome Measure

**Figure 1:**
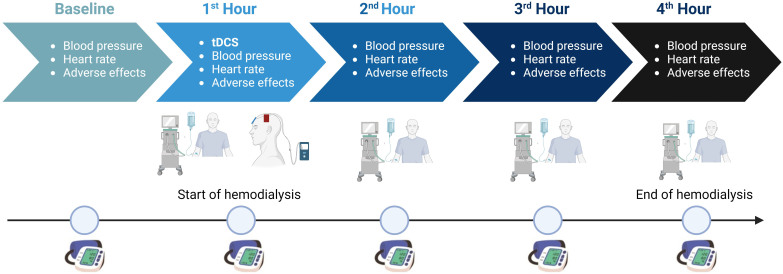
Details of hourly study evaluation and intervention process.

Clinical and sociodemographic information was assessed and included age, sex, body mass index, smoking, marital status, chronic kidney disease etiology, hemodialysis time, and comorbidities. A blinded experienced nephrologist performed all evaluative procedures. Systolic blood pressure, diastolic blood pressure, mean blood pressure, and heart rate were assessed before and during all ten sessions of tDCS at five time points: at baseline, one hour before the first tDCS session, and at the beginning of each four-hour hemodialysis session (Figure 1). The oscillometer method (Hem-7200, Omron, USA) was used to measure blood pressure and heart rate according to the European Society of Hypertension practice guidelines for office and out-of-office blood pressure measurement^15^.

### Randomization and Blinding

A computer randomized the patients in a 1:1 ratio (sham tDCS group or active tDCS group), according to their entry into the study. A researcher assistant not involved in the study generated the allocation sequence. The patients and the researcher involved in the assessments were therefore unaware of the patients’ allocation throughout the trial.

### Adverse Event Monitoring and Adherence

Adverse events were monitored through patient reporting during and after each tDCS session. Additionally, a nephrologist was present during the sessions to manage any adverse effects that might arise. During the hemodialysis procedure, various critical variables were monitored to ensure patient safety and optimize treatment outcomes. These variables included blood pressure, fluid balance, electrolyte levels, blood flow rate, dialysate flow rate and composition, temperature, and monitoring of patients’ subjective sensations and tolerance to tDCS. To improve participant adherence to the tDCS treatment protocol, the researchers offered an in-depth explanation of the potential benefits, which included the possibility of pain relief.

### Statistical Analysis

The software Jamovi (Version 2.3.28) was used to analyze the data. Data of quantitative variables are reported as means and standard deviations and data of qualitative variables, as percentages. The student’s t-test and Chi-square test was used to compare the baseline demographic characteristics and clinical scores between groups for continuous and categorical variables, respectively. For all times analyzed, systolic, diastolic, and mean blood pressure and heart rate presented the nonsymmetrical distribution known as Gamma distribution with link function identity. The independent factors were time, group, and interaction between them. The generalized mixed model was used to analyze the data before and after each of the ten sessions (every hour for four hours). The generalized mixed model with a random effect added to the constant of the model was used to identify individual variability. Data analysis is reported as mean difference and confidence interval, standard error, and p-value. Missing data was inputted using the group mean of each variable. The significance level was *p* < 5%.

## Results

Initially, a total of 62 patients were screened to participate in the study. Thirty-two patients did not meet the inclusion criteria or declined due to limitations. Thirty patients were randomized to the active or sham groups. There were no serious clinical complications related to the hemodialysis process among patients who completed the treatment protocols. There were only clinical events related to routine dialysis treatment, such as hypoglycemia, hypotension, cramps, headache, body pain, and hemodynamic instability. Table 1 shows the sample’s socio-demographic and clinical characteristics. There were no statistical differences between the groups before treatment for age, sex, body mass index, smoking, chronic kidney disease etiology, marital status, hemodialysis time, and comorbidities.

**Table 1:** Socio-demographic and clinical characteristics

Variables	Active group (n = 15)	Sham group (n = 15)	p value
Age	51.5 ± 12.0	56.7 ± 13.6	0.28
Sex (male) %	40	13	0.10
BMI	24.2 ± 5.10	25.1 ± 3.44	0.57
Smoking %	26	7	0.60
Marital Status %			0.10
Single	40	20	
Married	40	46	
Divorced	20	7	
Widow	0	27	
RCD etiology %			0.29
Chronic Glomerulonephritis	53	27	
Post-renal	7	0	
Hypertension	13	27	
Diabetes	27	46	
Hemodialysis time	85.8 ± 66.6	51.5 ± 41.3	0.10
Hypertension %	60	80	0.24
Diabetes %	26	73	0.26

Notes – Hemodialysis time in months. %: percentage. Continuous variables are reported as mean and standard deviation. Abbreviations – BMI: body mass index; RCD: renal chronic disease.

The patients tolerated the tDCS applications well. There were few adverse effects, such as headache or worsening of pre-existing headache (active: 0%; sham: 1.4%), nausea (active: 0%; sham: 0.7%), and tingling (active: 37.7%; sham: 0%) reported by participants during the 300 therapy sessions of the study. Any clinical events related to hemodialysis were treated according to the judgment of the attending physician, including hypoglycemia (glucose replacement), hypotension (pause in ultrafiltration and volume replacement), cramps (glucose or 20% sodium chloride replacement) and hemodynamic instability (pause in dialysis and implementation of measures for hemodynamic support).

There were no statistically significant differences in the between-group analysis at any time point. Considering all the 10 sessions, the groups presented differences in the mean (95% CI) for systolic –10.93 (–29.1; 7.2), diastolic –3.63 (–12.4; 5.1), and mean blood pressure –6.0 (–16.3; 4.2) and hear rate 2.26 (–2.5; 7.1).

**Figure 2:**
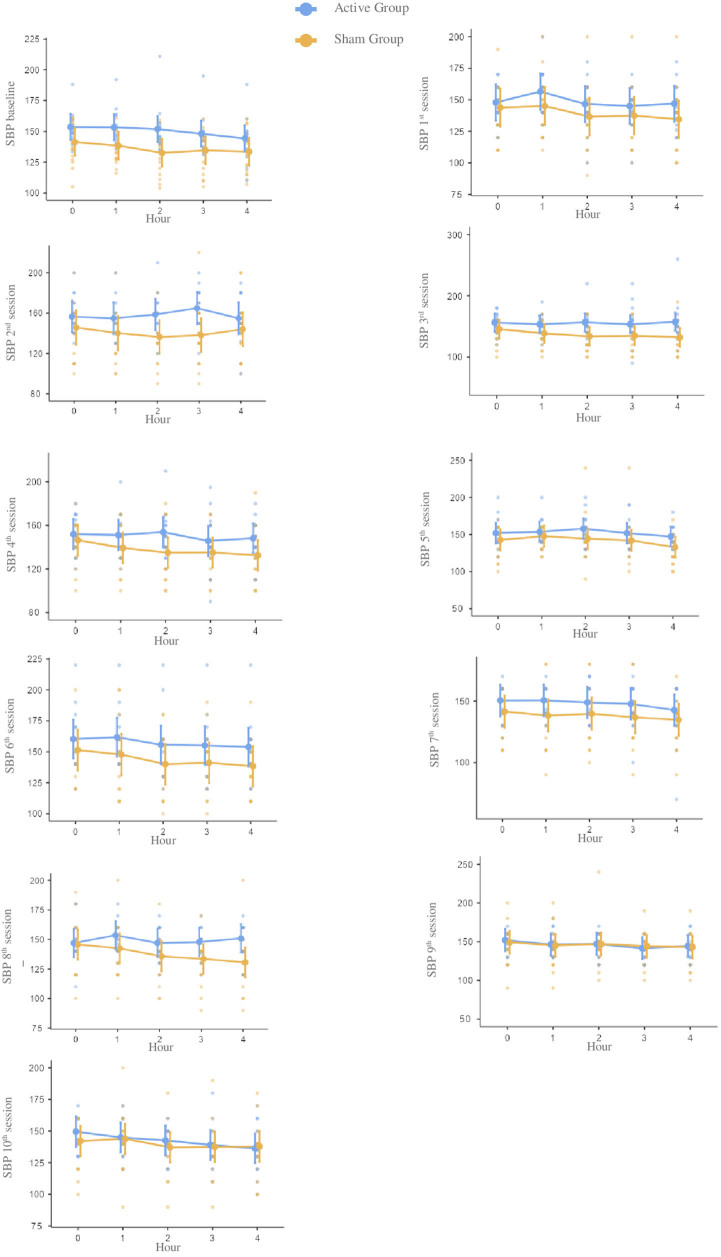
Notes – Mean scores and confidence intervals of systolic blood pressure at baseline and during each of the ten tDCS sessions during four hours. Each point represents an individual. Reference measure in millimeters of mercury. Abbreviation – SBP: systolic blood pressure.

**Figure 3:**
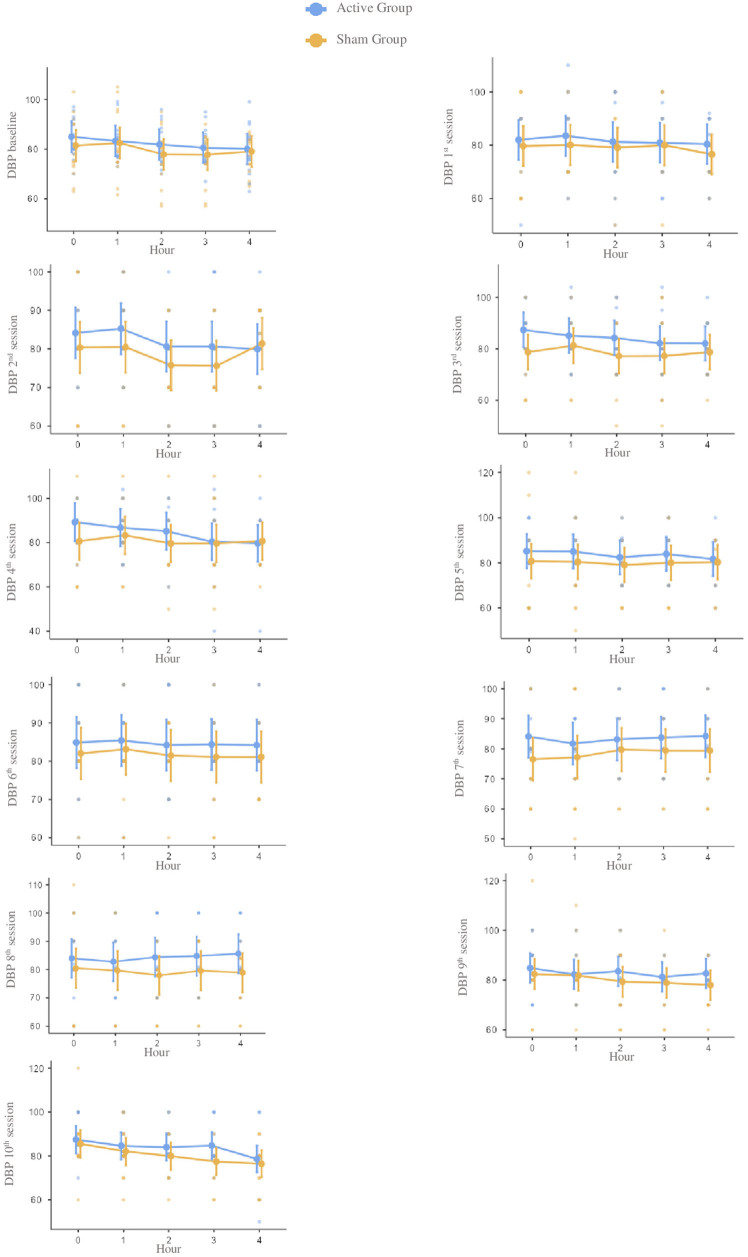
Notes – Mean scores and confidence intervals of diastolic blood pressure at baseline and during each of the ten tDCS sessions during four hours. Each point represents an individual. Reference measure in millimeters of mercury. Abbreviation – DBP: diastolic blood pressure.

**Figure 4:**
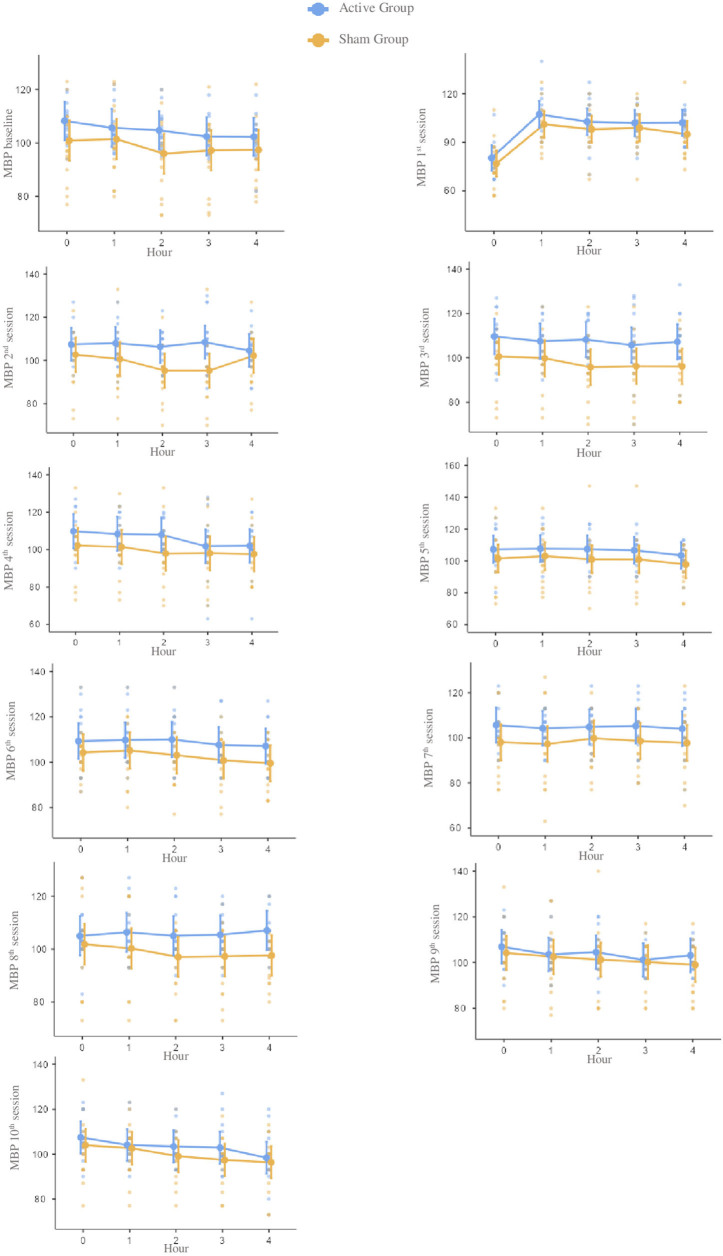
Notes – Mean scores and confidence intervals of mean blood pressure at baseline and during each of the ten tDCS sessions during four hours. Each point represents an individual. Reference measure in millimeters of mercury. Abbreviation – MBP: Mean blood pressure.

**Figure 5:**
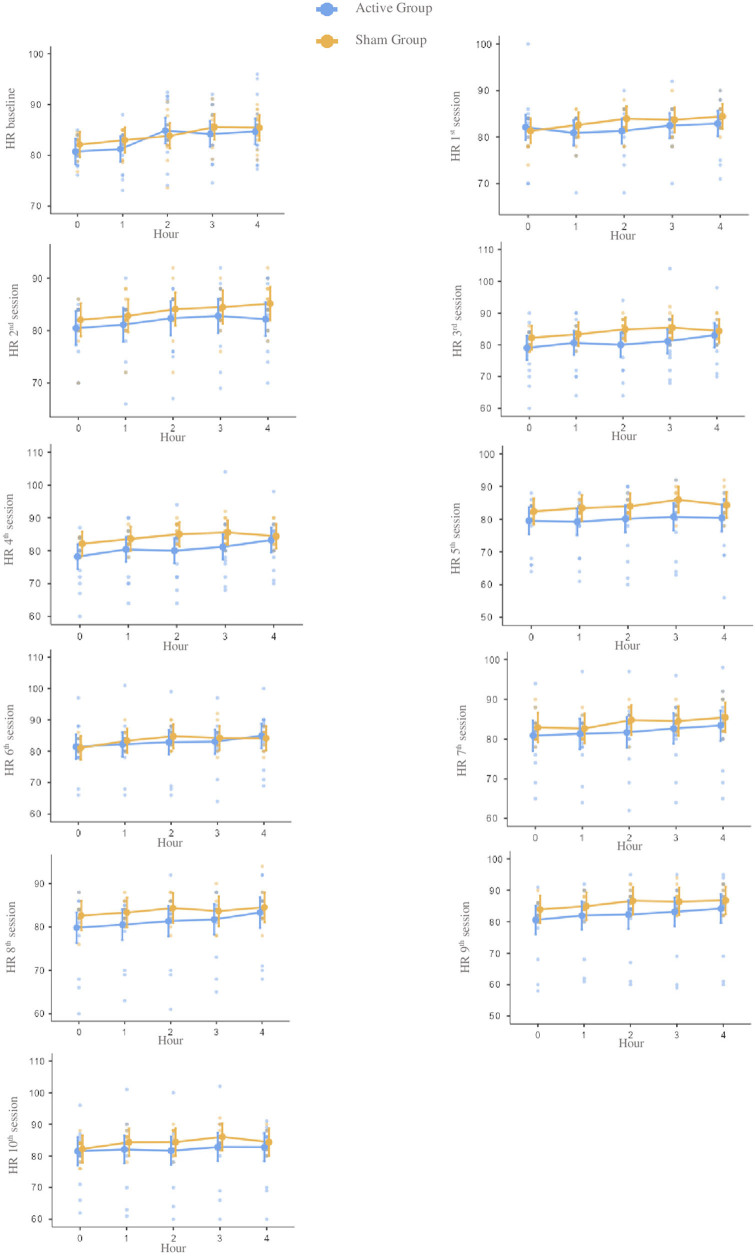
Notes – Mean scores and confidence intervals of heart rate at baseline and during each of the ten tDCS sessions during four hours. Each point represents an individual. Reference measure in beats per minute. Abbreviation – HR: heart rate.

As shown in Table 2, no statistical difference for systolic, diastolic, mean blood pressure, and heart rate were found between groups for all the moments before and after each stimulation session. However, the active group presented higher numeric values than the sham group for systolic, diastolic, and mean blood pressure (Figures 2, 3, 4 and Table 2). Also, heart rate values were higher in the sham group compared to the active group (Figure 5 and Table 2).

**Table 2:** Between-group analysis of hemodynamic variables in the five time points

Hemodynamic variables	Mean difference (CI)	Standard error	p value
Systolic blood pressure			
Baseline	–14.1 (–28.9; 0.6)	7.55	0.06
Session 1	–9.1 (–28.5; 10.3)	9.93	0.35
Session 2	–17.1 (–38.8; 4.6)	11.08	0.12
Session 3	–18.6 (–38.1; 0.8)	9.93	0.06
Session 4	–12.5 (–29.8; 4.7)	8.81	0.15
Session 5	–10.5 (–28.8; 7.7)	9.33	0.25
Session 6	–13.6 (–35.0; 7.7)	10.90	0.21
Session 7	–9.9 (–26.3; 6.4)	8.37	0.23
Session 8	–11.6 (–28.1; 4.9)	8.44	0.16
Session 9	–0.5 (–20.1; 19.0)	9.99	0.95
Session 10	–2.78 (–18.4; 12.9)	8.01	0.72
Diastolic blood pressure			
Baseline	–2.44 (–10.7; 5.8)	4.24	0.56
Session 1	–2.5 (–12.1; 7.0)	4.89	0.60
Session 2	–3.4 (–11.7; 4.9)	4.24	0.42
Session 3	–5.7 (–13.9; 2.8)	4.29	0.19
Session 4	–3.4 (–14.3; 7.3)	5.54	0.52
Session 5	–3.5 (–13.3; 6.2)	4.99	0.48
Session 6	–2.8 (–11.4; 5.7)	4.39	0.51
Session 7	–4.9 (–14.1; 4.3)	4.71	0.29
Session 8	–4.9 (–13.4; 3.5)	4.34	0.25
Session 9	–2.8 (–10.4; 4.7)	3.90	0.46
Session 10	–3.6 (–11.4; 4.2)	4.01	0.36
Mean blood pressure			
Baseline	–6.0 (–16.0; 3.9)	5.10	0.23
Session 1	–4.9 (–15.4; 5.6)	5.39	0.36
Session 2	–7.7 (–18.0; 2.5)	5.26	0.14
Session 3	–9.9 (–20.1; 0.2)	5.20	0.05
Session 4	–6.6 (–18.1; 4.9)	5.90	0.26
Session 5	–5.6 (–17.0; 5.6)	5.79	0.32
Session 6	–6.2 (–16.6; 4.2)	5.33	0.24
Session 7	–6.5 (–16.8; 3.8)	5.28	0.21
Session 8	–6.9 (–16.7; 2.7)	4.98	0.16
Session 9	–2.4 (–12.3; 7.4)	5.04	0.62
Session 10	–3.3 (–12.8; 6.0)	4.81	0.48
Heart rate			
Baseline	0.84 (–2.4; 4.0)	1.65	0.61
Session 1	1.2 (–2.1; 4.7)	1.76	0.47
Session 2	1.9 (–2.4; 6.2)	2.22	0.38
Session 3	3.2 (–1.6; 8.2)	2.51	0.19
Session 4	3.5 (–1.3; 8.4)	2.50	0.15
Session 5	4.0 (–1.5; 9.5)	2.82	0.15
Session 6	0.6 (–4.6; 5.8)	2.67	0.81
Session 7	2.0 (–3.1; 7.3)	2.68	0.43
Session 8	2.3 (–2.4; 7.1)	2.42	0.33
Session 9	3.3 (–2.8; 9.5)	3.16	0.29
Session 10	2.1 (–3.9; 8.2)	3.10	0.49

Note – Mean difference: sham group minus active group. Abbreviation – CI: 95% confidence interval.

## Discussion

This study showed that C3/Fp2 tDCS in patients with end-stage renal disease undergoing hemodialysis is safe and feasible. Moreover, there were no hemodynamic issues during or after the sessions of tDCS. It is emphasized that hypotension is frequently experienced by patients with end-stage renal disease undergoing hemodialysis^3^. So, it is possible that the higher values for blood pressure in the active group could be related to a hypotensive protective effect. However, more studies are needed to confirm this hypothesis.

As kidneys are responsible for modulating blood pressure and heart rate by different mechanisms,^16^ chronic kidney dysfunctions are frequently associated with hemodynamic alterations^16,17^. Therefore, during hemodialysis, systolic, diastolic, and mean blood pressure and heart rate must be carefully monitored. The use of different classes of medications is important to treat some symptoms and induce other systems to preserve homeostasis^18,19^. In an attempt to avoid or decrease the chronic use of medications to maintain hemodynamic functions and control pain, tDCS emerges as a therapeutic, safe, and feasible strategy^8^.

The central nervous system modulates peripheral vascular resistance, hormone release, heart rate, sympathetic and parasympathetic activity, and cardiac output^20,21,22^. Moreover, there is evidence that the modulation of the central nervous system improves hemodynamic variables in different populations^12^. This study showed that ten nonconsecutive sessions of tDCS did not significantly change the blood pressure and heart rate of patients with end-stage renal disease undergoing hemodialysis. However, the results suggested that the active group had a hypotensive protective effect . We spculate that the autonomic nervous system modulation is one of the mechanisms involved in this control^12^.

In addition, the heart rate values were numerically lower in the active group. The increase in parasympathetic activity and the decrease in sympathetic activity could be responsible for the reduction in the active group^23^. Therefore, the use of tDCS as a safe therapeutic strategy in patients with end-stage renal disease could improve homeostasis and medication efficacy, avoiding the overload and collateral damage of other systems.

It is important to mention that this study had limitations. The small number of patients may have increased the variability in the groups. Also, data analysis was not controlled for the drugs taken by the patients. However, as an exploratory study, tDCS was shown to be aviable an adjunctive strategy for patients with end-stage renal disease undergoing hemodialysis. Future studies with tDCS aiming to improve pain, physical function, mood, and quality of life could be performed with safety and feasibility.

## Conclusion

This trial suggests that ten non-consecutive sessions of tDCS are safe and feasible, taking into account the cardiovascular parameters of patients with end-stage renal disease undergoing hemodialysis. The observed adverse effects were similar to those reported in other tDCS studies, and no collateral effects were found. The potential cardiovascular protective effect of C3/Fp2 tDCS, achieved by modulating the central nervous system, should be considered.

## Data Availability

Available upon request.
