# Supplementary material for: Safety and feasibility of transcranial direct current stimulation in end-stage renal disease patients undergoing hemodialysis: an exploratory study
Source: J Bras Nefrol. 2024 Jul 19;46(4):e20240010. doi: 10.1590/2175-8239-JBN-2024-0010en (PMC11268524; doi:10.1590/2175-8239-JBN-2024-0010en)
Supplement: Translation

# Translation: Segurança e viabilidade da estimulação transcraniana por corrente contínua em pacientes com doença renal em estágio terminal submetidos à hemodiálise: um estudo exploratório

**DOI:** 10.1590/2175-8239-JBN-2024-0010pt

**Authors:** Rodrigo Pegado, Monaliza Melo, Tayanne Oehmen, Gianna Mastroianni Kirsztajn, Edson Silva-Filho, Artur Quintiliano

**Affiliations:** 1 Universidade Federal do Rio Grande do Norte, Programa de Pós-Graduação em Ciências da Reabilitação, Natal, RN, Brasil. Universidade Federal do Rio Grande do Norte Programa de Pós-Graduação em Ciências da Reabilitação Natal RN Brasil; 2 Universidade Federal do Rio Grande do Norte, Programa de Pós-Graduação em Ciências da Saúde, Natal, RN, Brasil. Universidade Federal do Rio Grande do Norte Programa de Pós-Graduação em Ciências da Saúde Natal RN Brasil; 3 Universidade Federal do Rio Grande do Norte, Natal, RN, Brasil. Universidade Federal do Rio Grande do Norte Natal RN Brasil; 4 Universidade Federal de São Paulo, Programa de Pós-Graduação em Nefrologia, São Paulo, SP, Brasil. Universidade Federal de São Paulo Programa de Pós-Graduação em Nefrologia São Paulo SP Brasil; 5 Universidade Federal do Rio Grande do Norte, Departamento de Medicina, Natal, RN, Brasil. Universidade Federal do Rio Grande do Norte Departamento de Medicina Natal RN Brasil

**Keywords:** Estimulação Transcraniana por Corrente Contínua, Diálise Renal, Pressão Arterial, Frequência Cardíaca

## Abstract

**Introdução:**

Pacientes com doença renal em estágio terminal (DRET) geralmente enfrentam uma rotina desafiadora de hemodiálise, restrições alimentares e diversos medicamentos, podendo afetar sua função hemodinâmica. Abordagens domiciliares, seguras e não farmacológicas, como a estimulação transcraniana por corrente contínua (ETCC), devem ser combinadas com tratamento convencional.

**Objetivo:**

Avaliar segurança e viabilidade da ETCC na pressão arterial e frequência cardíaca em pacientes com DRET em hemodiálise.

**Método:**

Estudo paralelo, randomizado, controlado por placebo. Foram incluídos pacientes em hemodiálise por mais de três meses. Os pacientes receberam dez sessões não consecutivas de ETCC de 2mA no córtex motor primário. Cada sessão durou 20 minutos. No início do estudo e após cada uma das dez sessões, a pressão arterial e frequência cardíaca dos pacientes foram medidas a cada hora durante quatro horas.

**Resultados:**

Trinta pacientes foram randomizados para grupo ativo ou sham. A diferença média entre grupos foi calculada como valor médio do grupo sham menos valor médio do grupo ativo. Apesar de não haver alterações estatísticas para todos os desfechos considerando as 10 sessões, encontramos diferenças entre os grupos para pressão arterial sistólica –10,93 (–29,1; 7,2), diastólica –3,63 (–12,4; 5,1) e média –6,0 (–16,3; 4,2) e frequência cardíaca 2,26 (–2,5; 7,1). Não encontramos eventos adversos graves. O grupo ativo apresentou valores maiores de pressão arterial em todos os pontos, enquanto a frequência cardíaca foi menor no grupo ativo.

**Conclusão:**

ETCC é segura e viável para pacientes com DRET submetidos à hemodiálise. Estudos futuros devem investigar se a ETCC pode potencialmente induzir um efeito hipotensor protetor durante a hemodiálise.

## Introdução

A doença renal em estágio terminal é uma consequência da doença renal crônica, considerada um problema global devido ao número crescente de indivíduos afetados e ao alto custo do tratamento^1^. Pesquisas estimam que a doença renal crônica afeta mais de 10% da população mundial. A incidência dos estágios mais graves, nos quais a hemodiálise é necessária, cresce anualmente a uma taxa de 6–7%^1^. No estágio grave, os pacientes são expostos a uma rotina semanal de múltiplas visitas a clínicas especializadas, medicamentos e limitações de atividade. Assim, pacientes com doença renal em estágio terminal apresentam dor crônica, limitações funcionais significativas, alterações na saúde mental e uma diminuição na qualidade de vida em geral^2^.

Um dos sintomas mais comuns apresentados por pacientes submetidos à hemodiálise são as oscilações hemodinâmicas, que muitas vezes são negligenciadas pela equipe de saúde^3^. Além das alterações hemodinâmicas, os sintomas associados são dor, depressão, ansiedade, síndrome das pernas inquietas e redução da qualidade do sono^4,5^. Nesse sentido, os tratamentos farmacológicos e não farmacológicos previnem e reduzem os sintomas físicos e comportamentais de pacientes com doença renal em estágio terminal submetidos à hemodiálise^4,5^. Entretanto, os efeitos colaterais relacionados aos medicamentos e à diálise podem aumentar a incidência de comorbidades e óbito^6,7^.

Recentemente, Quintiliano et al.^8^ apresentaram uma nova proposta terapêutica para pacientes com doença renal em estágio terminal submetidos à hemodiálise, com o objetivo de melhorar a dor, o humor e a função física geral. Os autores sugeriram que 10 sessões de estimulação transcraniana por corrente contínua (ETCC) anódica de 2 mA aplicadas no córtex motor primário (montagem C3/Fp2) melhoram a dor, a depressão, a ansiedade e a qualidade de vida de pacientes submetidos à hemodiálise^8^. A ETCC é uma técnica não invasiva para modular áreas cerebrais relacionadas à dor e ao humor^9,10^. Através de um fluxo de microcorrente, ocorre uma alteração na capacidade de despolarização neuronal e na plasticidade temporária dos circuitos neurais. Dependendo da configuração e da intensidade, são alcançados efeitos físicos e comportamentais^10^.

É importante mencionar que a maioria dos estudos se concentrou na resposta hemodinâmica após a ETCC em indivíduos saudáveis^11,12^. Além disso, os estudos não relataram a segurança hemodinâmica da ETCC durante procedimentos clínicos, incluindo a hemodiálise. Durante o procedimento de hemodiálise, os parâmetros hemodinâmicos podem apresentar variações clínicas significativas e gerar efeitos adversos. A instabilidade hemodinâmica durante a hemodiálise pode ocorrer em alguns pacientes e é uma possível complicação reconhecida do procedimento^13^. As complicações hemodinâmicas estão associadas a diferentes fatores, incluindo alterações no equilíbrio hídrico e eletrolítico, alterações na função cardíaca e hipotensão^13^.

A ETCC surge como uma ferramenta potencial de baixo custo para ajudar pacientes com doença renal em estágio terminal. É imperativo que a ETCC não interfira negativamente nos parâmetros hemodinâmicos durante ou após as sessões de hemodiálise. Além disso, a ETCC pode gerar efeitos protetores ao prevenir a hipotensão, que é frequentemente relatada por pacientes durante a hemodiálise. Portanto, nossa hipótese é que a aplicação da ETCC C3/Fp2 em pacientes com doença renal em estágio terminal durante a hemodiálise é segura e viável e pode prevenir disfunções hipotensivas. Considerando essas premissas, o estudo tem como objetivo avaliar a segurança e a viabilidade da ETCC em pacientes com doença renal em estágio terminal submetidos à hemodiálise e o impacto na pressão arterial e na frequência cardíaca.

## Métodos

### Desenho do Estudo

Este foi um ensaio de centro único, paralelo, randomizado, controlado por placebo, desenhado de acordo com a declaração *Consolidated Standards of Reporting Trials*^14^, a Declaração de Helsinque e a resolução nº 466/12 do Conselho Nacional de Saúde. Este estudo foi previamente aprovado pelo Comitê de Ética local (Faculdade de Ciências da Saúde de Trairí) (número 2715151) e registrado retrospectivamente no Registro Brasileiro de Ensaios Clínicos (RBR-46vhrkj). O estudo foi realizado no Instituto do Rim, Natal, Brasil, entre agosto de 2018 e fevereiro de 2020. Os pesquisadores explicaram o objetivo e o protocolo do estudo a todos os participantes, que precisaram assinar o consentimento informado por escrito para participar.

### Critérios de Elegibilidade

Os pacientes foram incluídos no estudo de acordo com os seguintes critérios: homens ou mulheres com idade entre 18 e 75 anos submetidos à hemodiálise (sessão de quatro horas) por mais de três meses e que apresentassem dor musculoesquelética crônica, cefaleia ou dor neuropática (pontuação >4 na escala visual analógica por mais de três meses). Foram excluídos os pacientes que apresentaram implantes elétricos no corpo, contraindicações clínicas para receber ETCC, como presença de metal no couro cabeludo ou cérebro, histórico de epilepsia ou convulsão, serem gestantes, apresentarem sinais de doença grave ou indicação de hospitalização, incluindo instabilidade hemodinâmica prévia, infarto agudo do miocárdio, infecção, acidente vascular cerebral e doença psiquiátrica.

### Intervenção

Um profissional de enfermagem experiente e treinado aplicou um total de dez sessões não consecutivas três vezes por semana (segunda, quarta e sexta-feira ou terça, quinta e sábado) em cada participante. Os pacientes iniciaram a hemodiálise e o monitoramento em uma cadeira confortável com suporte para as costas e braços. No início da hemodiálise, a ETCC foi montada e ligada.

Inicialmente, os eletrodos foram colocados em uma esponja de 35 cm^2^ hidratada com solução salina (154 mM de NaCl, aproximadamente 12 mL por esponja). Em seguida, o eletrodo anódico foi posicionado e fixado por faixas elásticas sobre a região do córtex motor primário esquerdo (C3) e o eletrodo catódico na região supraorbital direita (Fp2), de acordo com o sistema internacional de eletroencefalograma 10–20 na montagem “M1-SO”. No grupo ativo, a corrente direta começou com uma intensidade de 2 mA, iniciando com uma rampa *on* de corrente de 30 segundos. Após 20 minutos, uma rampa *off* de corrente de 30 segundos encerrou a sessão. O mesmo protocolo foi utilizado para o grupo sham, mas a rampa *on* e a rampa *off* da corrente ocorreram por apenas 30 segundos^8^. A corrente foi fornecida através de eletrodos por um estimulador alimentado por bateria, e a corrente foi verificada com um multímetro digital de precisão (DT832, WeiHua Electronic Co., Ltd, China) com um erro padrão de 1,5%. A aparência do dispositivo era idêntica nas configurações ativa e placebo. Por questões éticas, não houve alterações na rotina de hemodiálise (dias, horários e locais das sessões), medicamentos, exames laboratoriais e de imagem.

### Medida de Desfecho

Foram avaliadas informações clínicas e sociodemográficas, incluindo idade, sexo, índice de massa corporal, tabagismo, estado civil, etiologia da doença renal crônica, tempo de hemodiálise e comorbidades. Um nefrologista experiente, sem conhecimento do estudo, realizou todos os procedimentos de avaliação. A pressão arterial sistólica, a pressão arterial diastólica, a pressão arterial média e a frequência cardíaca foram avaliadas antes e durante todas as dez sessões de ETCC em cinco momentos: no início do estudo, uma hora antes da primeira sessão de ETCC e no início de cada sessão de hemodiálise de quatro horas (Figura 1). O método oscilométrico (Hem-7200, Omron, EUA) foi utilizado para medir a pressão arterial e a frequência cardíaca de acordo com as diretrizes práticas da Sociedade Europeia de Hipertensão para medição de pressão arterial em consultório e fora dele^15^.

**Figura 1:** Detalhes da avaliação do estudo e do processo de intervenção realizados por hora.

### Randomização e Cegamento

Um computador randomizou os pacientes em uma proporção de 1:1 (grupo ETCC sham ou grupo ETCC ativo), de acordo com sua entrada no estudo. Um pesquisador assistente não envolvido no estudo gerou a sequência de alocação. Portanto, os pacientes e o pesquisador envolvido nas avaliações não tinham conhecimento da alocação dos pacientes durante o ensaio.

### Monitoramento de Eventos Adversos e Adesão

Os eventos adversos foram monitorados por meio de relatos dos pacientes durante e após cada sessão de ETCC. Além disso, um nefrologista esteve presente durante as sessões para manejar quaisquer efeitos adversos que pudessem surgir. Durante o procedimento de hemodiálise, diversas variáveis críticas foram monitoradas para garantir a segurança do paciente e otimizar os desfechos do tratamento. Essas variáveis incluíram pressão arterial, equilíbrio hídrico, níveis de eletrólitos, taxa de fluxo sanguíneo, taxa de fluxo e composição do dialisato, temperatura e monitoramento das sensações subjetivas dos pacientes e tolerância à ETCC. Para melhorar a adesão dos participantes ao protocolo de tratamento com ETCC, os pesquisadores ofereceram uma explicação detalhada sobre os benefícios potenciais, que incluíam a possibilidade de alívio da dor.

### Análise Estatística

O software Jamovi (versão 2.3.28) foi utilizado para análise dos dados. Os dados das variáveis quantitativas são reportados como médias e desvios padrão e os dados das variáveis qualitativas, como porcentagens. O teste t de Student e o teste qui-quadrado foram usados para comparar as características demográficas basais e os escores clínicos entre os grupos para variáveis contínuas e categóricas, respectivamente. Para todos os períodos analisados, as pressões arterial sistólica, diastólica e média, bem como a frequência cardíaca, apresentaram a distribuição assimétrica conhecida como distribuição Gama com função de ligação identidade. Os fatores independentes foram tempo, grupo e a interação entre eles. O modelo misto generalizado foi utilizado para analisar os dados antes e depois de cada uma das dez sessões (a cada hora durante quatro horas). O modelo misto generalizado com efeito aleatório adicionado à constante do modelo foi usado para identificar a variabilidade individual. A análise dos dados é relatada como diferença média e intervalo de confiança, erro padrão e valor de p. Os dados ausentes foram inseridos usando a média do grupo de cada variável. O nível de significância foi *p* < 5%.

## Resultados

Inicialmente, um total de 62 pacientes foi selecionado para participar do estudo. Trinta e dois pacientes não preencheram os critérios de inclusão ou recusaram a participação devido a limitações. Trinta pacientes foram randomizados para os grupos ativo ou sham. Não houve complicações clínicas graves relacionadas ao processo de hemodiálise entre os pacientes que completaram os protocolos de tratamento. Houve apenas eventos clínicos relacionados ao tratamento de diálise de rotina, como hipoglicemia, hipotensão, cãibras, cefaleia, dor no corpo e instabilidade hemodinâmica. A Tabela 1 mostra as características sociodemográficas e clínicas da amostra. Não houve diferenças estatísticas entre os grupos antes do tratamento para idade, sexo, índice de massa corporal, tabagismo, etiologia da doença renal crônica, estado civil, tempo de hemodiálise e comorbidades.

**Tabela 1:** Características sociodemográficas e clínicas

Variáveis	Grupo ativo (n = 15)	Grupo sham (n = 15)	Valor de p
Idade	51,5 ± 12,0	56,7 ± 13,6	0,28
Sexo (masculino) %	40	13	0,10
IMC	24,2 ± 5,10	25,1 ± 3,44	0,57
Tabagismo %	26	7	0,60
Estado Civil %			0,10
Solteiro	40	20	
Casado	40	46	
Divorciado	20	7	
Viúvo	0	27	
Etiologia DRC %			0,29
Glomerulonefrite Crônica	53	27	
Pós-renal	7	0	
Hipertensão	13	27	
Diabetes	27	46	
Tempo de hemodiálise	85,8 ± 66,6	51,5 ± 41,3	0,10
Hipertensão %	60	80	0,24
Diabetes %	26	73	0,26

Notas – Tempo de hemodiálise em meses. %: porcentagem. Variáveis contínuas são relatadas como média e desvio padrão. Abreviações – IMC: índice de massa corporal; DRC: doença renal crônica.

Os pacientes toleraram bem as aplicações de ETCC. Houve poucos efeitos adversos, como cefaleia ou piora da cefaleia preexistente (ativo: 0%; sham: 1,4%), náusea (ativo: 0%; sham: 0,7%) e formigamento (ativo: 37,7%; sham: 0%) relatados pelos participantes durante as 300 sessões de terapia do estudo. Quaisquer eventos clínicos relacionados à hemodiálise foram tratados de acordo com o julgamento do médico assistente, incluindo hipoglicemia (reposição de glicose), hipotensão (pausa na ultrafiltração e reposição volêmica), cãibras (reposição de glicose ou cloreto de sódio a 20%) e instabilidade hemodinâmica (pausa na diálise e implementação de medidas de suporte hemodinâmico).

Não houve diferenças estatisticamente significativas na análise entre grupos em nenhum momento. Considerando todas as 10 sessões, os grupos apresentaram diferenças na média (IC 95%) para pressão arterial sistólica –10,93 (–29,1; 7,2), diastólica –3,63 (–12,4; 5,1) e média –6,0 (–16,3; 4,2) e frequência cardíaca 2,26 (–2,5; 7,1).

Como mostra a Tabela 2, não houve diferença estatística para pressão arterial sistólica, diastólica, média e frequência cardíaca entre os grupos em todos os momentos antes e após cada sessão de estimulação. Entretanto, o grupo ativo apresentou valores numéricos mais elevados do que o grupo sham para pressão arterial sistólica, diastólica e média (Figuras 2, 3, 4 e Tabela 2). Além disso, os valores de frequência cardíaca foram superiores no grupo placebo em comparação com o grupo ativo (Figura 5 e Tabela 2).

**Tabela 2:** Análise entre grupos das variáveis hemodinâmicas nos cinco períodos de tempo

Variáveis hemodinâmicas	Diferença média (IC)	Erro padrão	Valor de p
Pressão arterial sistólica			
Basal	–14,1 (–28,9; 0,6)	7,55	0,06
Sessão 1	–9,1 (–28,5; 10,3)	9,93	0,35
Sessão 2	–17,1 (–38,8; 4,6)	11,08	0,12
Sessão 3	–18,6 (–38,1; 0,8)	9,93	0,06
Sessão 4	–12,5 (–29,8; 4,7)	8,81	0,15
Sessão 5	–10,5 (–28,8; 7,7)	9,33	0,25
Sessão 6	–13,6 (–35,0; 7,7)	10,90	0,21
Sessão 7	–9,9 (–26,3; 6,4)	8,37	0,23
Sessão 8	–11,6 (–28,1; 4,9)	8,44	0,16
Sessão 9	–0,5 (–20,1; 19,0)	9,99	0,95
Sessão 10	–2,78 (–18,4; 12,9)	8,01	0,72
Pressão arterial diastólica			
Basal	–2,44 (–10,7; 5,8)	4,24	0,56
Sessão 1	–2,5 (–12,1; 7,0)	4,89	0,60
Sessão 2	–3,4 (–11,7; 4,9)	4,24	0,42
Sessão 3	–5,7 (–13,9; 2,8)	4,29	0,19
Sessão 4	–3,4 (–14,3; 7,3)	5,54	0,52
Sessão 5	–3,5 (–13,3; 6,2)	4,99	0,48
Sessão 6	–2,8 (–11,4; 5,7)	4,39	0,51
Sessão 7	–4,9 (–14,1; 4,3)	4,71	0,29
Sessão 8	–4,9 (–13,4; 3,5)	4,34	0,25
Sessão 9	–2,8 (–10,4; 4,7)	3,90	0,46
Sessão 10	–3,6 (–11,4; 4,2)	4,01	0,36
Pressão arterial média			
Basal	–6,0 (–16,0; 3,9)	5,10	0,23
Sessão 1	–4,9 (–15,4; 5,6)	5,39	0,36
Sessão 2	–7,7 (–18,0; 2,5)	5,26	0,14
Sessão 3	–9,9 (–20,1; 0,2)	5,20	0,05
Sessão 4	–6,6 (–18,1; 4,9)	5,90	0,26
Sessão 5	–5,6 (–17,0; 5,6)	5,79	0,32
Sessão 6	–6,2 (–16,6; 4,2)	5,33	0,24
Sessão 7	–6,5 (–16,8; 3,8)	5,28	0,21
Sessão 8	–6,9 (–16,7; 2,7)	4,98	0,16
Sessão 9	–2,4 (–12,3; 7,4)	5,04	0,62
Sessão 10	–3,3 (–12,8; 6,0)	4,81	0,48
Frequência cardíaca			
Basal	0,84 (–2,4; 4,0)	1,65	0,61
Sessão 1	1,2 (–2,1; 4,7)	1,76	0,47
Sessão 2	1,9 (–2,4; 6,2)	2,22	0,38
Sessão 3	3,2 (–1,6; 8,2)	2,51	0,19
Sessão 4	3,5 (–1,3; 8,4)	2,50	0,15
Sessão 5	4,0 (–1,5; 9,5)	2,82	0,15
Sessão 6	0,6 (–4,6; 5,8)	2,67	0,81
Sessão 7	2,0 (–3,1; 7,3)	2,68	0,43
Sessão 8	2,3 (–2,4; 7,1)	2,42	0,33
Sessão 9	3,3 (–2,8; 9,5)	3,16	0,29
Sessão 10	2,1 (–3,9; 8,2)	3,10	0,49

Nota – Diferença média: grupo sham menos grupo ativo. Abreviação – IC: intervalo de confiança de 95%.

**Figura 2:** Notas – Scores médios e intervalos de confiança da pressão arterial sistólica no início do estudo e durante cada uma das dez sessões de ETCC por quatro horas. Cada ponto representa um indivíduo. Medida de referência em milímetros de mercúrio. Abreviações – PAS: Pressão arterial sistólica.

**Figura 3:** Notas – Scores médios e intervalos de confiança da pressão arterial diastólica no início do estudo e durante cada uma das dez sessões de ETCC por quatro horas. Cada ponto representa um indivíduo. Medida de referência em milímetros de mercúrio. Abreviação – PAD: Pressão arterial diastólica.

**Figura 4:** Notas – Scores médios e intervalos de confiança da pressão arterial média no início do estudo e durante cada uma das dez sessões de ETCC por quatro horas. Cada ponto representa um indivíduo. Medida de referência em milímetros de mercúrio. Abreviação – PAM: Pressão arterial média.

**Figura 5:** Notas – Scores médios e intervalos de confiança da frequência cardíaca no início do estudo e durante cada uma das dez sessões de ETCC por quatro horas. Cada ponto representa um indivíduo. Medida de referência em batimentos por minuto. Abreviação – FC: frequência cardíaca.

## Discussão

Este estudo mostrou que a ETCC C3/Fp2 em pacientes com doença renal em estágio terminal submetidos à hemodiálise é segura e viável. Além disso, não houve problemas hemodinâmicos durante ou após as sessões de ETCC. É importante ressaltar que a hipotensão é frequentemente vivenciada por pacientes com doença renal em estágio terminal submetidos à hemodiálise^3^. Portanto, é possível que os valores mais elevados de pressão arterial no grupo ativo possam estar relacionados a um efeito protetor hipotensor. No entanto, são necessários mais estudos para confirmar essa hipótese.

Como os rins são responsáveis por modular a pressão arterial e a frequência cardíaca por diferentes mecanismos^16^, as disfunções renais crônicas estão frequentemente associadas a alterações hemodinâmicas^16,17^. Portanto, durante a hemodiálise, a pressão arterial sistólica, diastólica e média e a frequência cardíaca devem ser cuidadosamente monitoradas. O uso de diferentes classes de medicamentos é importante para tratar alguns sintomas e induzir outros sistemas a preservar a homeostase^18,19^. Na tentativa de evitar ou diminuir o uso crônico de medicamentos para manter as funções hemodinâmicas e controlar a dor, a ETCC surge como uma estratégia terapêutica, segura e viável^8^.

O sistema nervoso central modula a resistência vascular periférica, a liberação de hormônios, a frequência cardíaca, a atividade simpática e parassimpática e o débito cardíaco^20–22^. Além disso, há evidências de que a modulação do sistema nervoso central melhora as variáveis hemodinâmicas em diferentes populações^12^. Esse estudo mostrou que dez sessões não consecutivas de ETCC não alteraram significativamente a pressão arterial e a frequência cardíaca de pacientes com doença renal em estágio terminal submetidos à hemodiálise. No entanto, os resultados sugeriram que o grupo ativo teve um efeito protetor hipotensor. Especulamos que a modulação do sistema nervoso autônomo seja um dos mecanismos envolvidos nesse controle^12^.

Além disso, os valores de frequência cardíaca foram numericamente mais baixos no grupo ativo. O aumento na atividade parassimpática e a diminuição na atividade simpática podem ser responsáveis pela redução no grupo ativo^23^. Portanto, o uso da ETCC como estratégia terapêutica segura em pacientes com doença renal em estágio terminal poderia melhorar a homeostase e a eficácia da medicação, evitando a sobrecarga e os danos colaterais de outros sistemas.

É importante mencionar que este estudo teve limitações. O pequeno número de pacientes pode ter aumentado a variabilidade nos grupos. Além disso, a análise dos dados não foi controlada quanto aos medicamentos tomados pelos pacientes. No entanto, como um estudo exploratório, a ETCC demonstrou ser uma estratégia adjuvante viável para pacientes com doença renal em estágio terminal submetidos à hemodiálise. Estudos futuros com ETCC com o objetivo de melhorar a dor, a função física, o humor e a qualidade de vida podem ser realizados com segurança e viabilidade.

## Conclusão

Esse estudo sugere que dez sessões não consecutivas de ETCC são seguras e viáveis, considerando os parâmetros cardiovasculares de pacientes com doença renal em estágio terminal submetidos à hemodiálise. Os efeitos adversos observados foram semelhantes aos relatados em outros estudos de ETCC e não foram encontrados efeitos colaterais. O potencial efeito protetor cardiovascular da ETCC C3/Fp2, obtido por meio da modulação do sistema nervoso central, deve ser considerado.

## Data Availability

Disponíveis mediante solicitação.
